# Emergency imaging in paediatric oncology: a pictorial review

**DOI:** 10.1186/s13244-019-0796-5

**Published:** 2019-12-18

**Authors:** Trevor Gaunt, Felice D’Arco, Anne M. Smets, Kieran McHugh, Susan C. Shelmerdine

**Affiliations:** 1 Great Ormond Street Hospital for Children NHS Foundation Trust, London, WC1N 3JH UK; 2 Academic Medical Center, PO Box 22700, Amsterdam, 1100 DE The Netherlands; 3 UCL Great Ormond Street Institute of Child Health, London, UK

**Keywords:** Emergency, Neoplasms, Child, Radiology, Tumour

## Abstract

Despite the decline in mortality rates over the last 20 years, cancer remains one of the leading causes of death in children worldwide. Early recognition and treatment for acute oncological emergencies are vital in preventing mortality and poor outcomes, such as irreversible end-organ damage and a compromised quality of life.

Imaging plays a pivotal and adjunctive role to clinical examination, and a high level of interpretative acumen by the radiologist can make the difference between life and death. In contrast to adults, the most accessible cross-sectional imaging tool in children typically involves ultrasound. The excellent soft tissue differentiation allows for careful delineation of malignant masses and along with colour Doppler imaging, thromboses and large haematomas can be easily identified. Neurological imaging, particularly in older children is an exception. Here, computed tomography (CT) is required for acute intracranial pathologies, with magnetic resonance imaging (MRI) providing more definitive results later.

This review is divided into a ‘body systems’ format covering a range of pathologies including neurological complications (brainstem herniation, hydrocephalus, spinal cord compression), thoracic complications (airway obstruction, superior vena cava syndrome, cardiac tamponade), intra-abdominal complications (bowel obstruction and perforation, hydronephrosis, abdominal compartment syndrome) and haematological-related emergencies (thrombosis, infection, massive haemorrhage). Within each subsection, we highlight pertinent clinical and imaging considerations.

The overall objective of this pictorial review is to illustrate how primary childhood malignancies may present with life-threatening complications, and emphasise the need for imminent patient management.

## Key points

- Paediatric cancers differ from adult malignancies in their type, prevalence and location and therefore knowledge of tumour behaviours is crucial in predicting acute complications.
- Conventional radiography can be helpful in suspected bowel perforation, bowel obstruction or airway obstruction from mediastinal masses; however, ultrasound and CT are frequently more informative.
- MR imaging, whilst excellent for characterising and staging a primary malignancy, has less of a role in the emergency scenario apart from in neurological emergencies, such as suspected spinal cord compression.

## Background

Cancer remains one of the leading causes of death in children, after 1 year of age [1], despite the vast improvement in cancer treatment. Over the last 20 years, the overall 5-year survival rates now reach approximately 80% [2–4]. Nevertheless, increased awareness and rapid assessment of acute oncological-related emergencies could further reduce poor outcomes. Whilst paediatric malignancies are comparatively rarer than in the adult population, they are biologically very different and consist of differing tumour types which predispose to differing types of emergency presentation [5]. Many emergency physicians have little experience of paediatric oncological emergencies and may feel ill-equipped in such complex scenarios to understand the extent of the underlying medical and mechanical issues faced [6]. Given that imaging plays a pivotal role in these scenarios, the radiologist is in a privileged position whereby a high level of interpretative acumen can make the difference between life and death.

This pictorial review serves to illustrate the many facets and scenarios seen during emergency presentations of paediatric cancers. We outline the abnormalities and best imaging modalities for each scenario by body systems and include cases seen at presenting diagnosis, prior to curative surgical resection and unrelated to chemotoxic agents. Acute presentations relating to complications of treatment and infective diseases are not included, given their substantial coverage elsewhere in the literature [7–10].

## Neurological emergencies

### Raised intracranial pressure

**Fig. 1:**
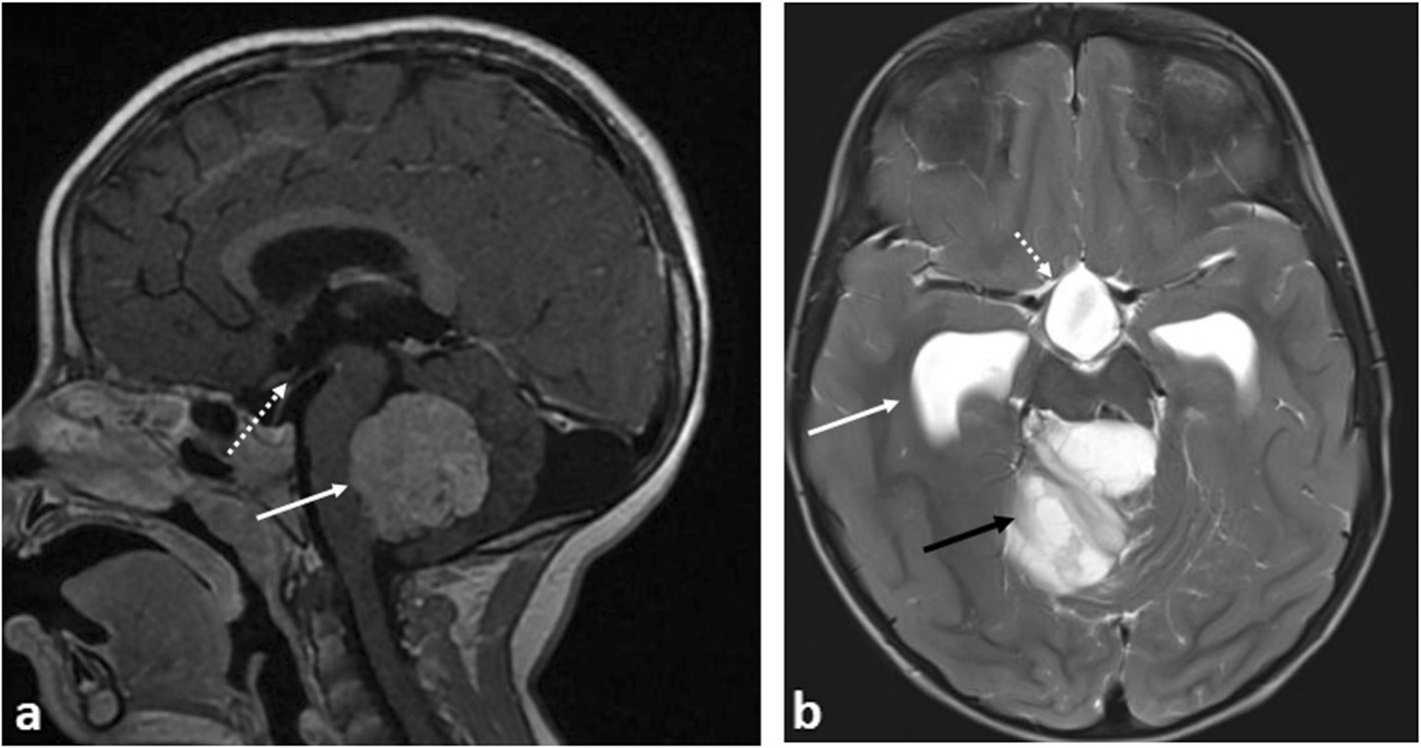
Three examples of causes of obstructive hydrocephalus in different patients due to different intracranial tumours. **a** Sagittal post-contrast T1-weighted MRI image showing a medulloblastoma in the fourth ventricle with mass effect on the brainstem (solid white arrow). There are no early signs of hydrocephalus—the floor of the third ventricle is not bulging inferiorly (dashed white arrow)—despite the compression of the brainstem. **b** Axial T2-weighted MRI image in patient with a pilocytic astrocytoma in the posterior fossa (black arrow) with signs of hydrocephalus and dilatation of the temporal horns (solid white arrow) of the lateral ventricles and inferior dilatation of the third ventricle (dashed white arrow)

Tumours of the central nervous system are the leading cause of cancer-related deaths in children [11], with the majority located in the infratentorium [12]. Both low- and high-grade tumours may cause acute hydrocephalus either from extrinsic compression or intraventricular extension of the tumour adjacent to the foramen of Monroe, cerebral aqueduct, fourth ventricle and outlet foramina [13]. The commonest low-grade tumour in the posterior fossa includes pilocytic astrocytomas whilst high-grade tumours are divided into ependymomas and embryonal tumours (such as medulloblastomas, atypical terato-rabdoid tumours (ATRT) and embryonal tumours with multi-layered rosettes (ETMR)) [12]. (Fig. 1a, b) In the emergency setting however, determining the tumour subtype is not the main goal, which should be directed towards identification and localisation of the mass, any adverse complications (e.g. hydrocephalus or haemorrhage) and aiding the neurologists and neurosurgeons in planning subsequent clinical interventions. This may include the insertion of an extraventricular drain (EVD) in the first instance, to help relieve the hydrocephalus, occasionally to drain haemorrhage and provide ongoing intracranial pressure (ICP) monitoring [14]. One large case series of approximately 180 children found that an underlying cerebral neoplasm was the primary indication for an EVD in almost a third (32.2%) of cases, with astrocytomas accounting for 43.1% of all responsible tumours [13].

Presenting symptoms can be variable depending on age and commonly non-specific in nature. On the whole, intracranial malignancies have a subacute onset and commonly present with changes in mood, headache or intermittent seizures [15]. In rare instances, such as during a neurological emergency, a primary tumour or intracranial metastasis may present with symptoms of raised ICP and hydrocephalus due to obstruction (such as from a posterior fossa mass [12]) or overproduction (e.g. from choroid plexus papilloma) of cerebrospinal fluid (CSF). In certain cases, the interval increase in perilesional oedema or intra-tumoural haemorrhage may be the precipitating event leading to the acute emergency [15]. Late clinical signs of a raised intracranial pressure may lead to the Cushing’s triad (reduced respiratory rate, bradycardia, systolic hypertension) as well as seizures.

Focal neurology and stroke-like symptoms are more likely from tumour haemorrhage or venous infarction from cerebral venous sinus thrombosis (CVST) which although rare, has been associated with childhood intracranial malignancies [17]. In one review by Sebire et al., 4% of children presenting with CVST had an underlying brain tumour at presentation, with the sagittal and transverse sinuses most commonly involved [18].

Imaging protocols for acute intracranial oncological pathologies are similar to those in children without suspected malignancies. Cranial ultrasound is a quick and accessible screening test in patients with patent fontanelles. Ventricular dilatation, a mass lesion or focally abnormal parenchymal echotexture in the context of stroke or haemorrhage, can be easily and quickly demonstrated. Colour Doppler can easily assess the superior sagittal sinus for thrombosis. Unenhanced computed tomography (CT) allows for rapid identification of intracranial shift, haemorrhage, oedema and hydrocephalus, and enhanced CT (in the form of CT venography) can be used to demonstrate deep sinus thrombosis or arterial compromise. Ultimately, neurosurgical consultation should be obtained, with magnetic resonance imaging (MRI) used for further lesion characterisation [19], particularly of posterior fossa masses which are better depicted with MR. Fortunately, brainstem herniation is a rare occurrence, but children presenting with these features should be imaged with the most definitive modality available, which in most cases will be MRI.

### Cord compression and cauda equina syndrome

**Fig. 2:**
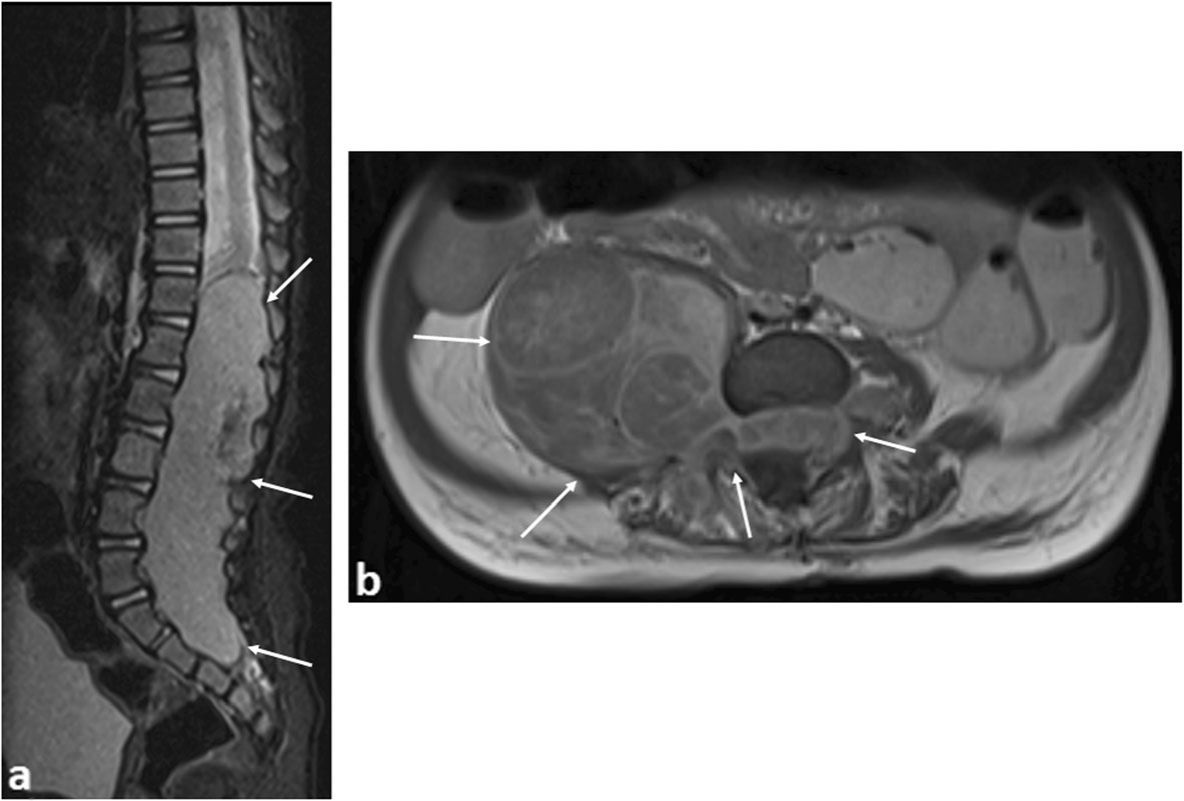
A 9-month-old boy with paravertebral dumbbell neuroblastoma. **a** Sagittal T2-weighted fat-saturated MR image shows a large soft tissue mass occupying and expanding the spinal canal from T12 to S3 levels (arrows), also causing compression of the spinal cord. **b** Axial T1 post-contrast imaging reveals a large right paravertebral mass (arrows) with intraspinal extension, occupying the entire right-sided neural foramen. Urgent spinal laminectomy and decompression was subsequently performed

Acute spinal cord compression occurs in 3–5% of children with cancer at diagnosis [20], usually from external compression by a paravertebral tumour (Fig. 2), commonly presenting with back pain [21]. Prolonged compression can progress to irreversible neurological damage within hours [22].

Potential causative lesions commonly include neuroblastomas (with 5–15% of such cases having spinal canal involvement [23] at diagnosis) and less frequently soft tissue sarcomas (Ewing’s sarcoma or rhabdomyosarcoma), Hodgkin’s lymphoma and primary spinal cord tumours (such as ependymomas, astrocytomas and intraspinal chloromas in acute myeloid leukaemia).

**Fig. 3:**
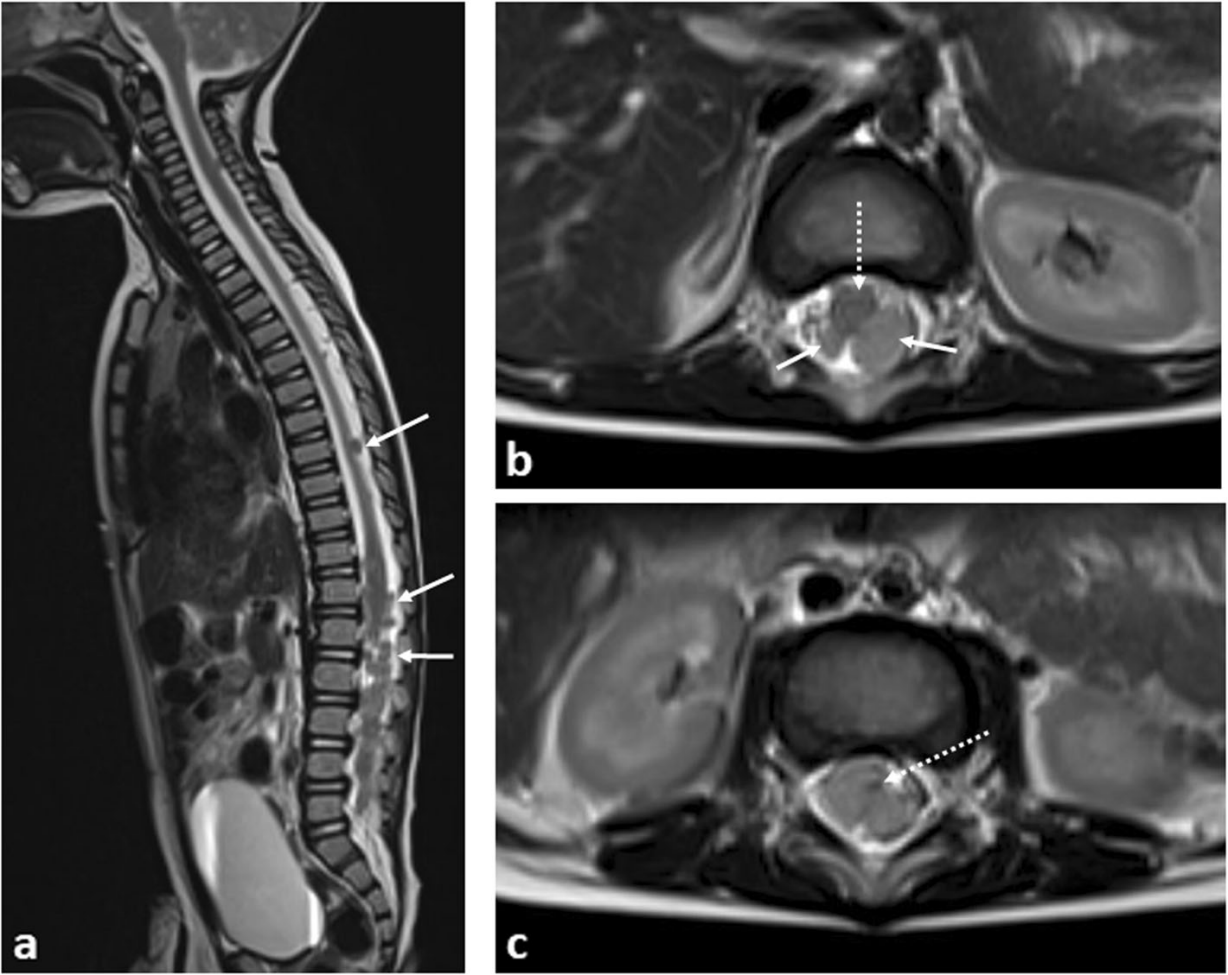
A 9-month-old boy with disseminated spinal leptomeningeal disease secondary to an intracranial ATRT. **a** Sagittal T2-weighted MRI of the spine demonstrates numerous extra-medullary, intraspinal tumour deposits (arrows). **b** Axial T2-weighted MRI of the lumbar spine demonstrate how these deposits (arrows) occupy the majority of the spinal canal, with the spinal cord (dashed arrow) anteriorly displaced, and (**c**) eventually becoming compressed at the cauda equina, where it is barely visible (dashed arrow). Emergency intrathecal chemotherapy and radiotherapy was subsequently performed

Rarely, metastatic disease affecting the leptomeninges of the spine can occur. Whilst this heralds a poor outcome in adults, the prognosis in children is better, and early identification can allow for a more considerate and careful planning of chemotherapy and, rarely, adjuvant radiotherapy treatments. In recent years, improvements in survival have been shown with proton beam therapy [24] (Fig. 3).

In the above scenarios, plain radiographs of the spine are generally unhelpful. MRI imaging is the ideal modality and should include the entire neuraxis (the brain and whole spine), with sedation and analgesia occasionally required to allow for adequate patient positioning. Following the initial imaging of the brain, an additional dose of gadolinium contrast agent is not required—the dose provided for post-contrast brain imaging is normally sufficient for the spine given that both body areas are usually imaged within the same sitting, lasting less than 1hour in length [25].

### Proptosis

**Fig. 4:**
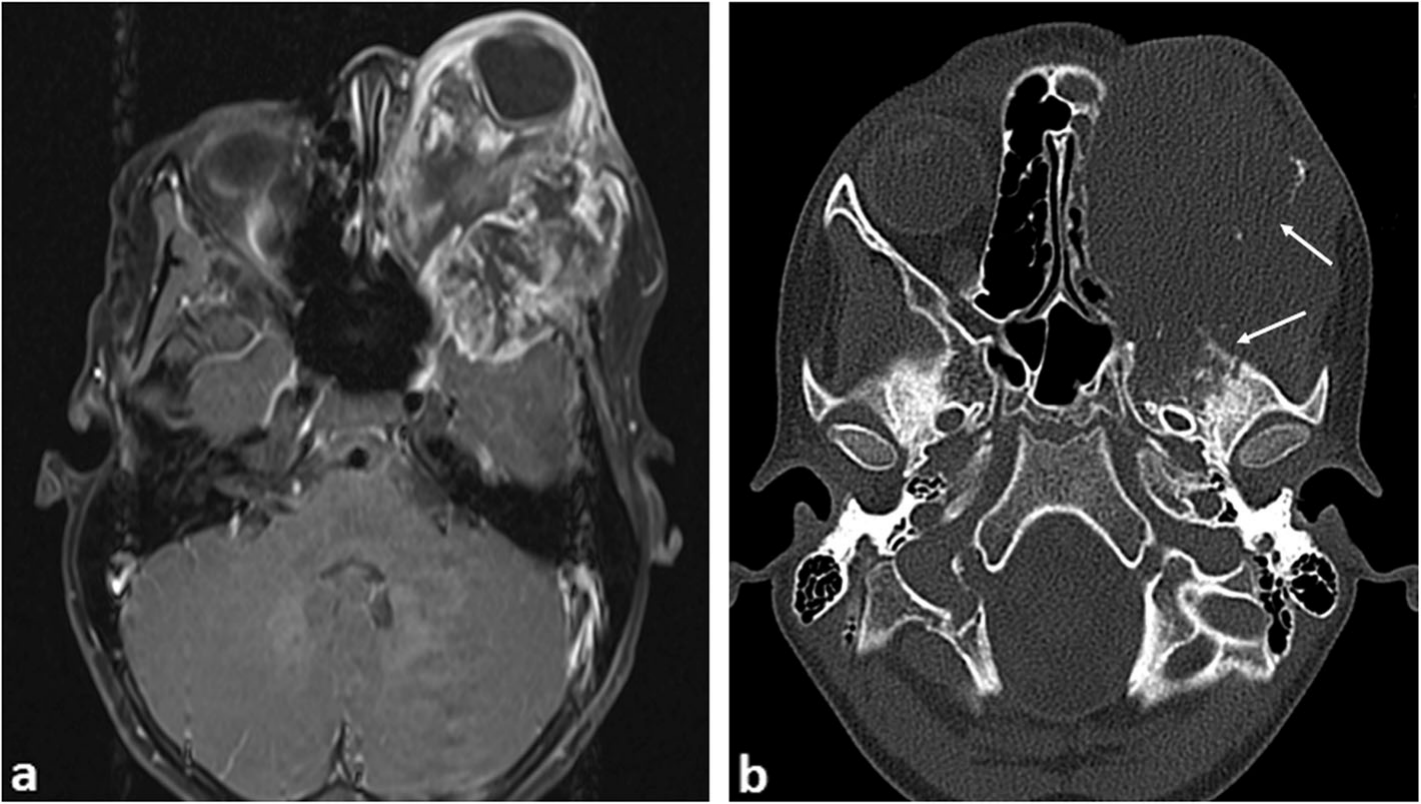
A 9-year-old boy with proptosis due to a left orbital rhabdomyosarcoma. **a** Axial post-contrast-enhanced T1-weighted MR image shows a large periorbital soft tissue mass with marked internal heterogenous enhancement invading the temporal fossa. **b** Axial CT image of the orbit demonstrates marked bony destruction of the skull and left ethmoid sinuses (arrows)

**Fig. 5:**
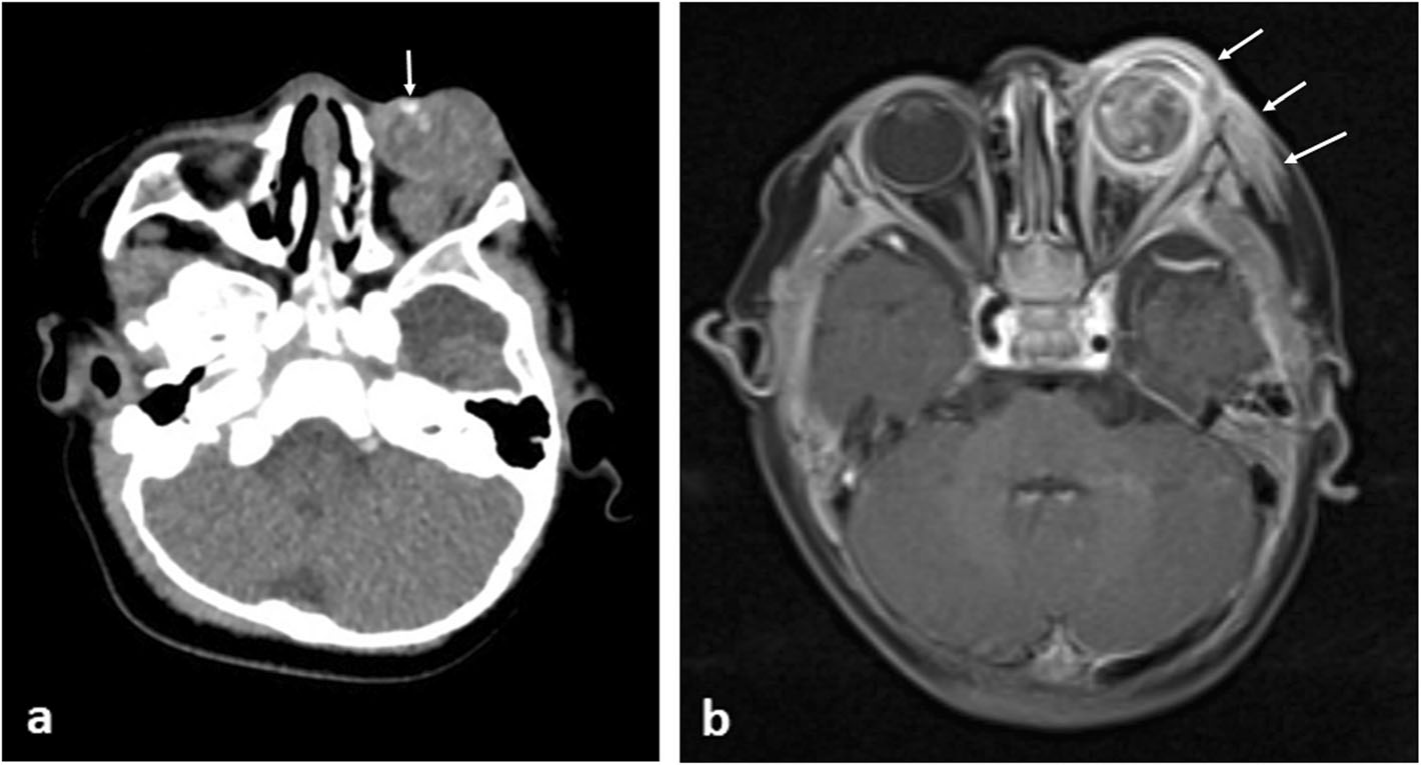
A 6-month-old male with biopsy proven intraocular rhabdoid tumour. **a** Axial CT showing a large left intraorbital mass involving mainly the intraconal compartment with associated marked proptosis. The lesion demonstrates internal calcifications (arrow). **b** Post-contrast fat-saturated T1-weighted MR image shows intra and periorbital abnormal contrast enhancement (arrows)

Causes of proptosis include benign and malignant pathologies. Orbital cellulitis is often clinically apparent with CT being used to confirm post-septal extension. MRI examination through the orbits is often definitive for benign aetiologies, with orbital haemangiomas and pseudotumour having characteristic MR appearances [26]. The most common primary malignancy to present with proptosis is orbital rhabdomyosarcoma (RMS) and confers a high risk of blindness if diagnosis is delayed (Fig. 4) [27]. RMS accounts for 4% of all paediatric malignancies, with 10% occurring in the orbit, most frequently within the first decade. If the primary lesion is less than 5 cm and embryonal-type histologically, local staging with MRI is often sufficient. If larger than 5 cm or alveolar-type, full-body PET-CT should be performed [28]. Causes of orbital metastases include malignant rhabdoid tumour (MRT) (Fig. 5) which typically presents with rapidly progressive proptosis in infants [29, 30]. As such, MR imaging should include the orbits and neuraxis, as synchronous primary and secondary intracranial neoplasms are seen in 15% of cases [31]. In malignant rhaboid tumours, the kidney is the most frequent site of origin with metastases to the lungs being common. Therefore further work-up should also include imaging of these body systems.

## Intra-thoracic emergencies

### Airway compression and SVC syndrome

**Fig. 6:**
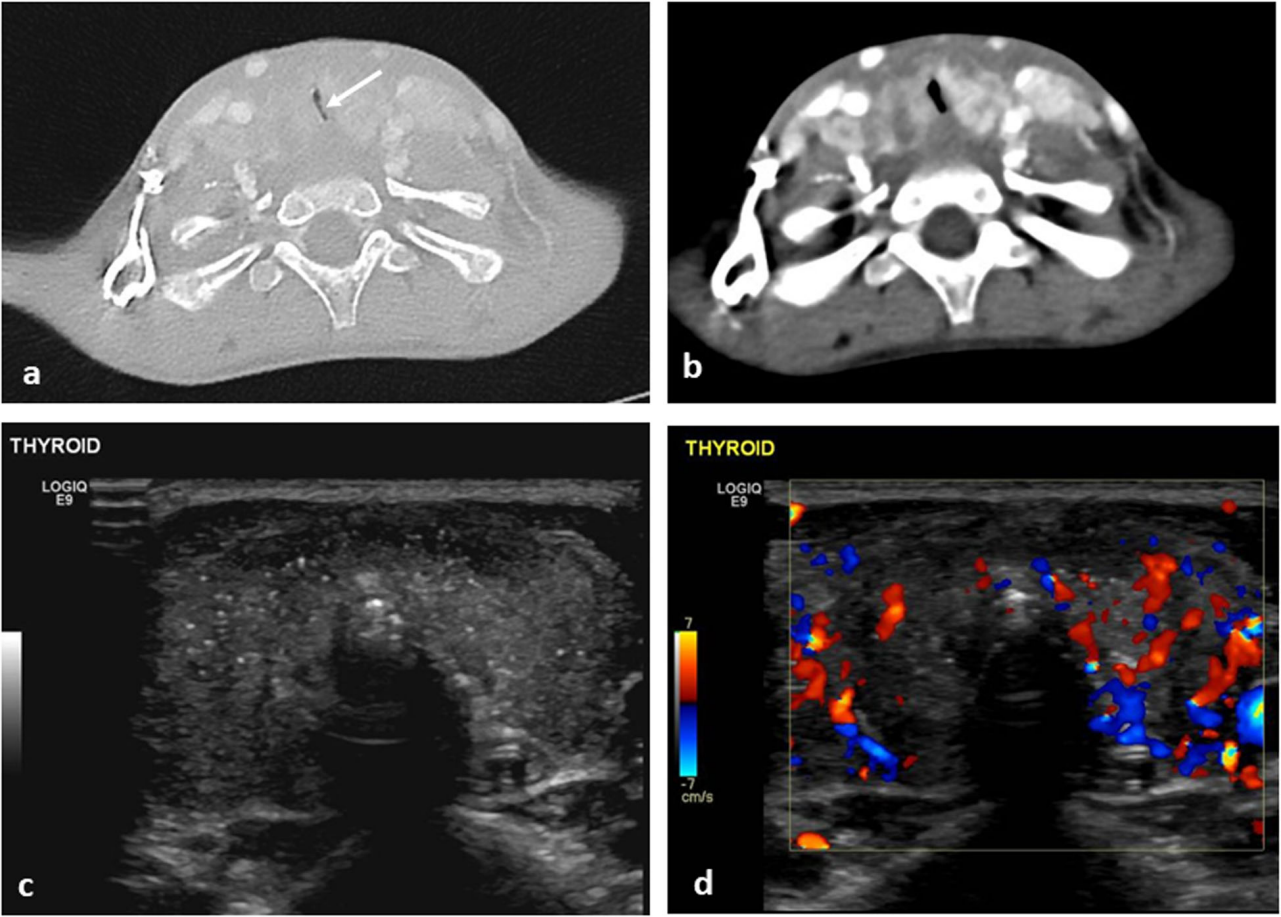
A 5-year-old boy with worsening stridor, dry cough and cervical lymphadenopathy with papillary thyroid carcinoma. **a** Axial CT of the neck (lung windows) demonstrates marked narrowing of the trachea, with slit-like appearance (arrow). **b** On the mediastinal windows, the thyroid gland is bulky and demonstrates patchy heterogenous enhancement. **c** Transverse ultrasound image of the thyroid gland prior to biopsy reveals multiple internal foci of calcification and (**d**) colour Doppler imaging reveals a hyper vascular thyroid gland

The smaller diameter and compressible nature of the trachea in children places them at increased risk of airway obstruction with anterior mediastinal masses. Common aetiologies include lymphoma, leukaemia and germ cell tumours. These tumours may also cause vessel compression [32] resulting in superior vena cava (SVC) syndrome. Thyroid tumours may also cause airway compression but they are rare in children (Fig. 6).

Common symptoms of airway obstruction include cough, stridor and dyspnoea [33], with poor correlation between the severity of symptoms and degree of airway obstruction [34]. Where SVC syndrome is present, children may present with cardiogenic shock from reduced venous return [35].

**Fig. 7:**
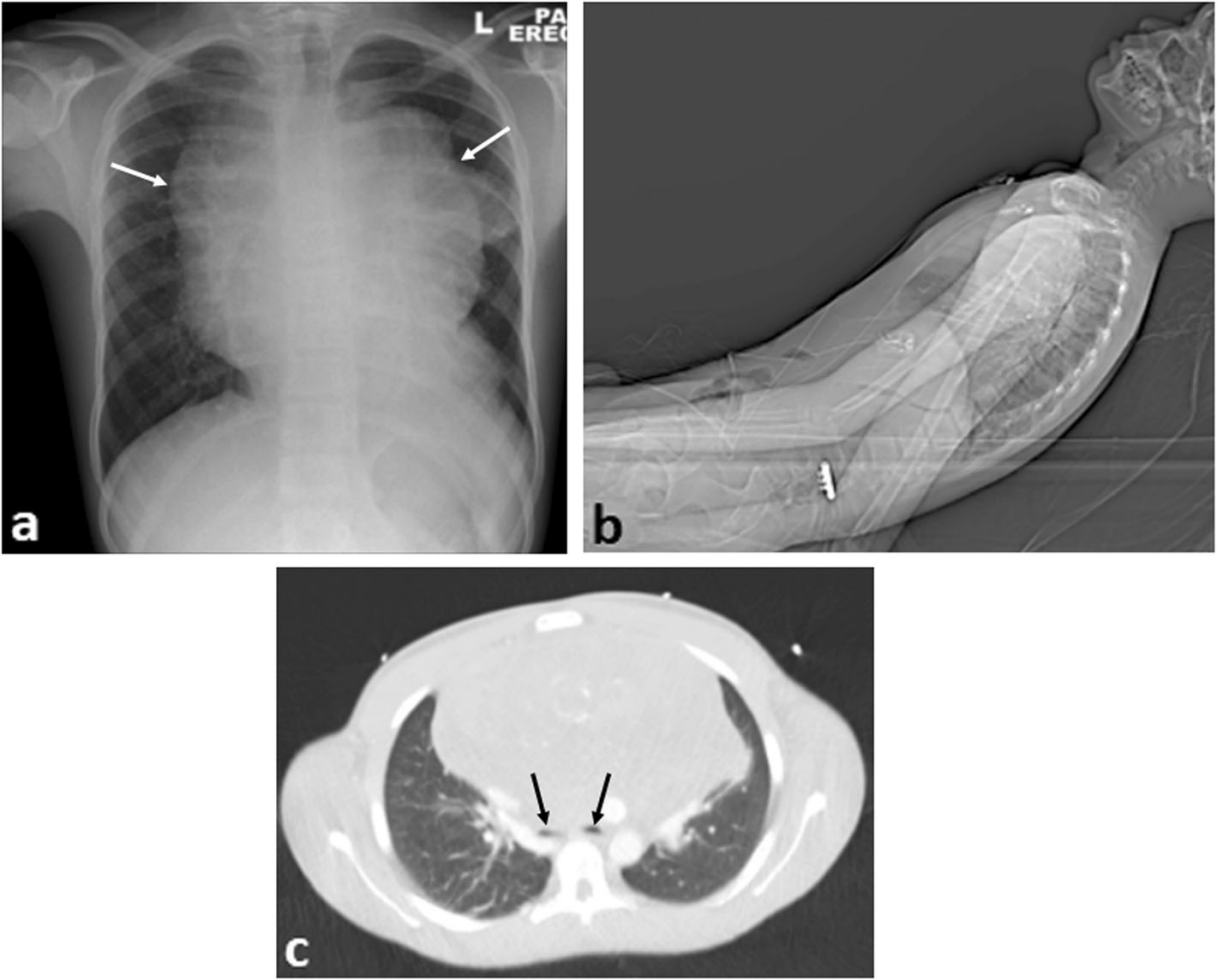
A 15-year-old boy with difficulty breathing and stridor presented with a large anterior mediastinal mass secondary to Hodgkin’s lymphoma. **a** Erect plain chest radiograph reveals a widened mediastinum (arrows). **b** Lateral view of the topogram from the CT scout image demonstrates the semi-upright positioning of the patient in the scanner because of reduced air entry on lying supine. **c** Axial CT imaging (lung windows) at the carina demonstrates marked airway compression of the main bronchi (black arrows) from the anterior mediastinal mass

**Fig. 8:**
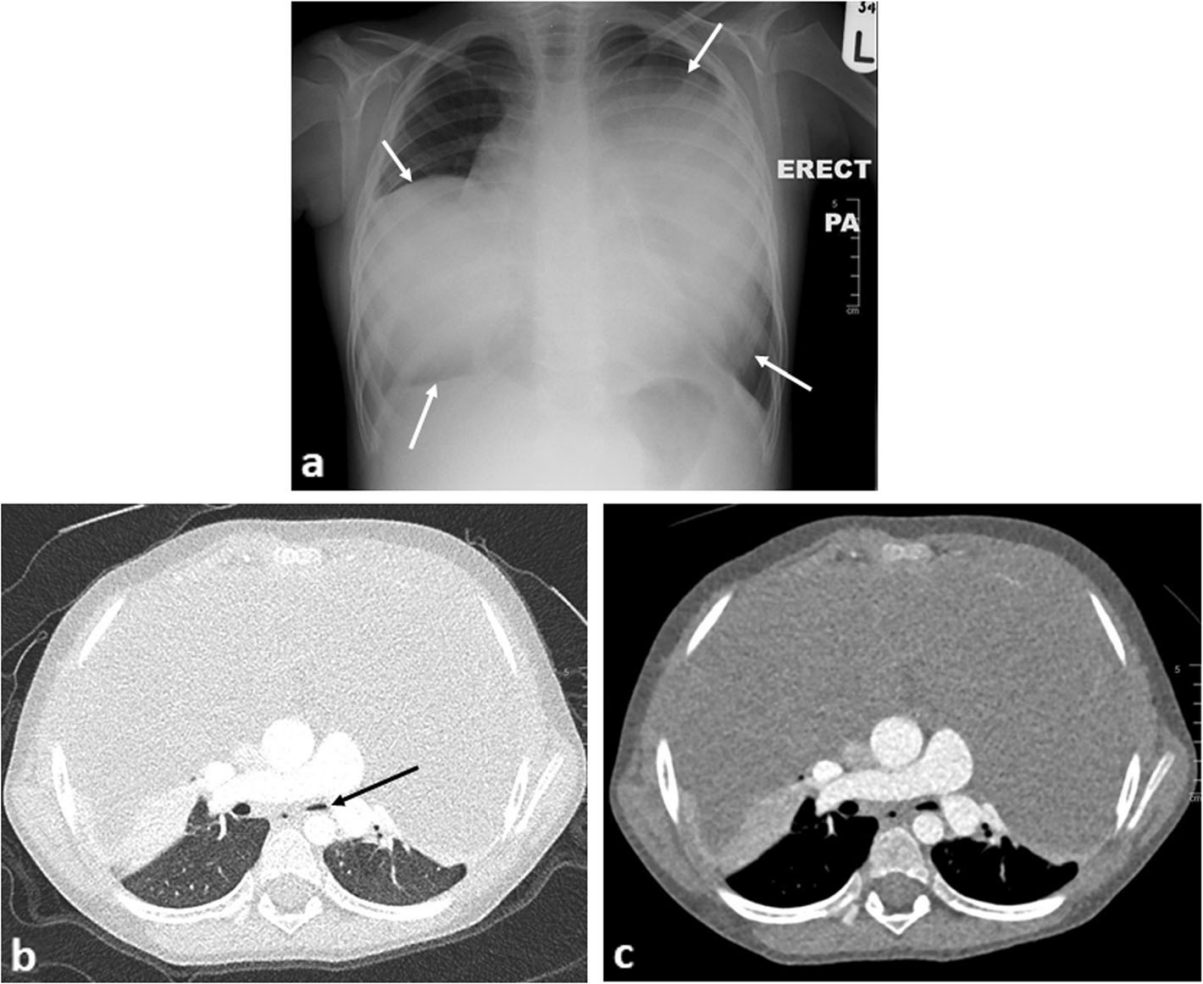
A 4-year-old boy presenting with shortness of breath secondary to desmoid fibromatosis (a benign, non-cancerous tumour). **a** The erect chest radiograph demonstrates an enlarged anterior mediastinum with bilateral lobular mass-like appearances (arrows). **b** Axial contrast-enhanced CT of the chest (lung windows) demonstrates some compression of the left lower lobe bronchus (black arrow), with patency of the right lower lobe bronchus. **c** Axial CT image of the chest (mediastinal windows) demonstrates the homogenous nature of the anterior mediastinal mass (which would be unusual in a suspected teratoma). In contrast to the previous figure, this patient was able to lie flat in the scanner for their imaging study despite the seemingly larger sized mass on imaging. This demonstrates how imaging findings may not necessarily relate to the patient’s symptoms and careful assessment and history remain vital

Plain chest radiographs identify mediastinal masses in 97% of cases, if present [36] and aid assessment of tracheal narrowing. CT or MR imaging will further characterise the mass but may not be practical where there is positional compromise to breathing. In such cases, imaging may need to be obtained with the patient in a semi-upright or prone position (Figs. 7 and 8).

Maintaining a calm presence, minimal child handling and obtaining a tissue diagnosis via the least invasive method possible is recommended [37]. General anaesthesia should only be used if absolutely necessary, with one study finding that those with a tracheal cross-sectional area of < 30% of normal (‘normal’ defined as widest tracheal diameter at the thoracic inlet, with lung apices visible on axial slice) or with the addition of bronchial compression being associated with a greater risk of intra and post-operative complications [38–41].

**Fig. 9:**
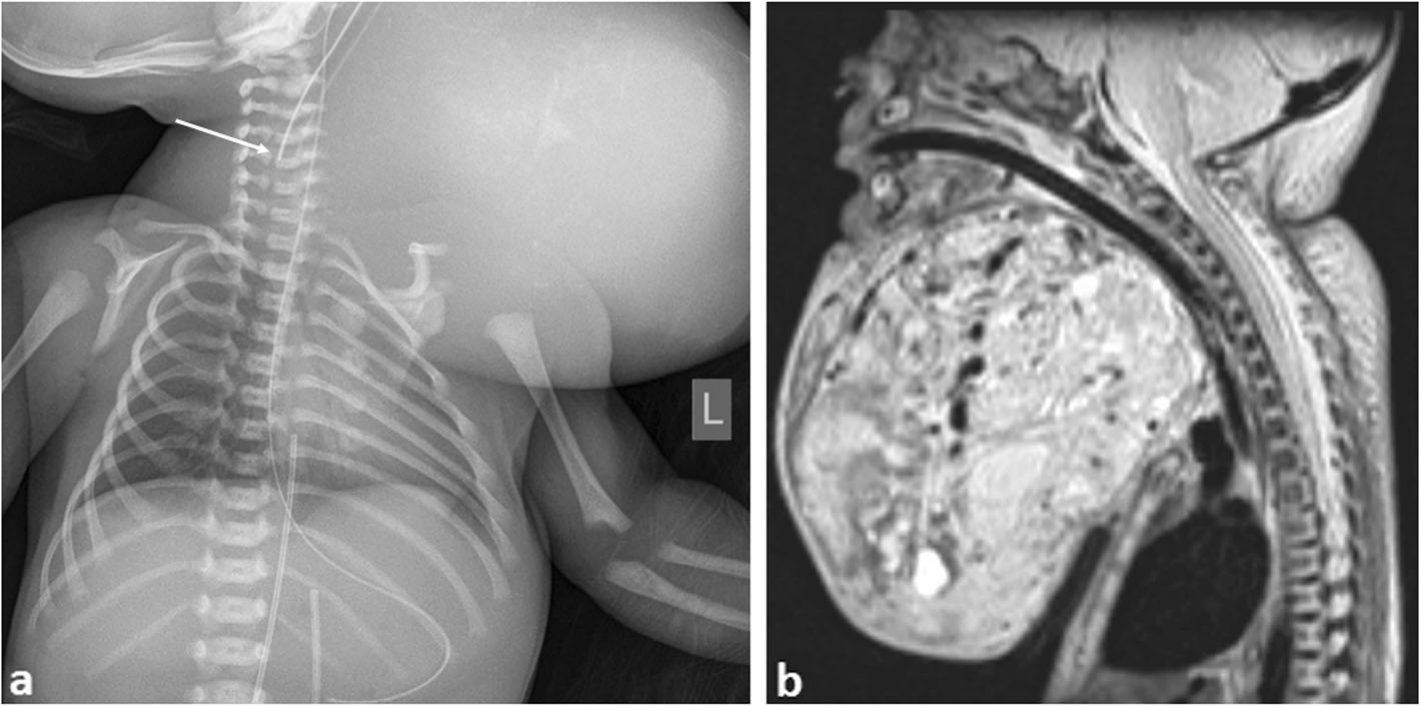
A neonate antenatally diagnosed with a large cervical teratoma. The patient was delivered at 34 weeks gestation by ex utero intrapartum treatment (EXIT) procedure due to impending airway obstruction. **a** Initial chest radiograph of patient post intubation. Note the high position of the endotracheal tube tip from difficult intubation (arrow). This should lie at the T2 vertebral level. **b** Sagittal postnatal T2-weighted MRI image demonstrating the large cervical mass and proximity to the upper airway. The patient had been successfully intubated during the MRI study, thus accounting for the patency of upper airway diameter in this image

In the antenatal period, obstetric sonography may identify a potentially fatal airway obstruction from a large neck mass, commonly a cervical teratoma [42] (Fig. 9). Although this is unlikely to present as an acute emergency, careful planning and identification of the relationship between the tumour and adjacent airway and vascular anatomy is crucial. Antenatal MRI has recently been hailed as the most definitive imaging tool for these cases [43], allowing for surgical planning of an ‘ex-utero intrapartum treatment’ (‘EXIT’) of the baby, followed by definitive surgery to remove the tumour. Given the location and potential airway complications in such cases, successful long-term outcomes are usually achieved in just over half of cases [44].

### Pleural effusion

Although malignancy is a rare cause, pleural effusion may be seen at diagnosis in 15% of children with non-Hodgkin lymphoma and is a marker for poor therapeutic response and relapse. Suggested causes include venous obstruction from bulky disease, leading to a decreased venous return to the heart and reduced lymphatic drainage of the thoracic duct if the left brachiocephalic vein is involved [45]. Pleural effusions are readily demonstrated on chest radiography and pleural ultrasound, with the latter able to assess the complexity, and approximate size of the effusion. Furthermore, ultrasound may be used to identify an appropriate site for surgical drain placement or in image-guided percutaneous drainage [46].

### Pericardial effusion and tamponade

**Fig. 10:**
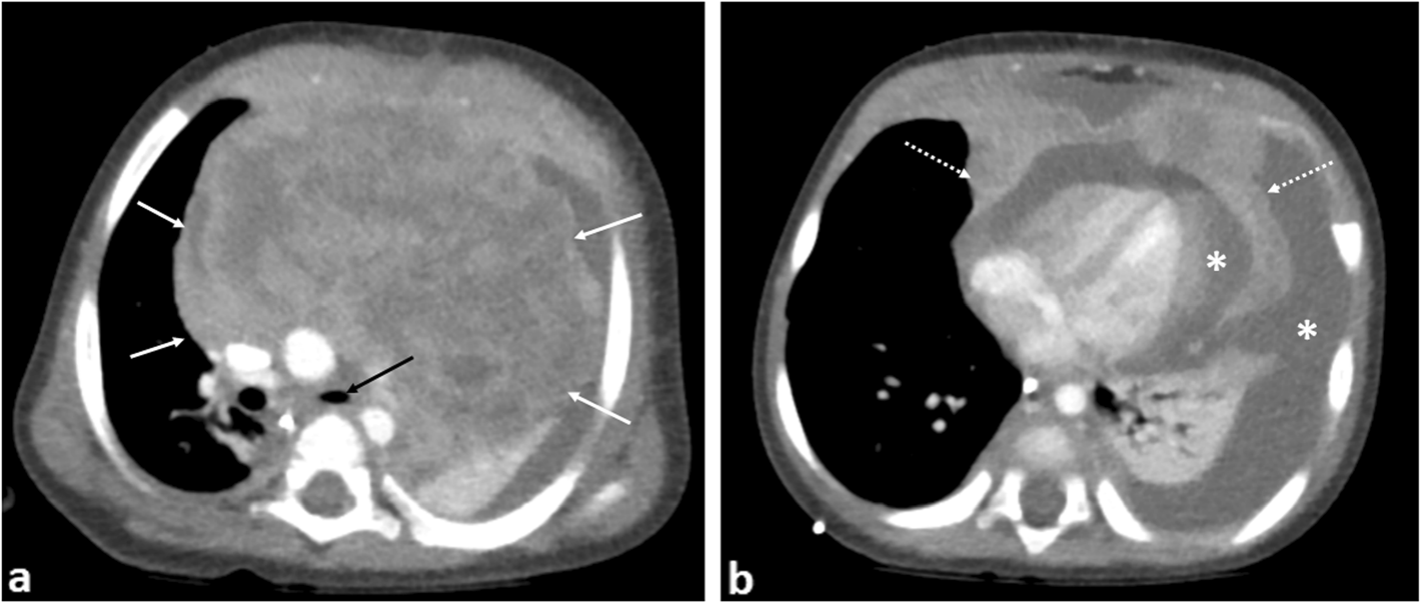
An 8-month-old boy with T-cell lymphoblastic lymphoma. **a** Contrast-enhanced axial CT image of the superior mediastinum and (**b**) at the level of the cardiac ventricles demonstrate a large heterogeneously enhancing anterior mediastinal mass (white arrows) with pericardial invasion (dashed arrows). There is narrowing of the left main bronchus (black arrow), left lung collapse and a large pericardial and left pleural effusion (asterisks)

Causes of pericardial effusion in paediatric oncology patients are myriad ranging from general medical causes of cardiac failure, side effects of chemotherapy, radiotherapy or haematopoietic stem-cell transplantation [47] to malignant pericardial effusions. The latter is a rare complication associated with pericardial infiltration from leukaemia and lymphoma [48, 49], intrapericardial [50] or primary cardiac tumours [51] (Fig. 10). Urgent cardiology consultation with echocardiography and a view to proceeding to pericardiocentesis is essential.

### Diaphragmatic splinting

**Fig. 11:**
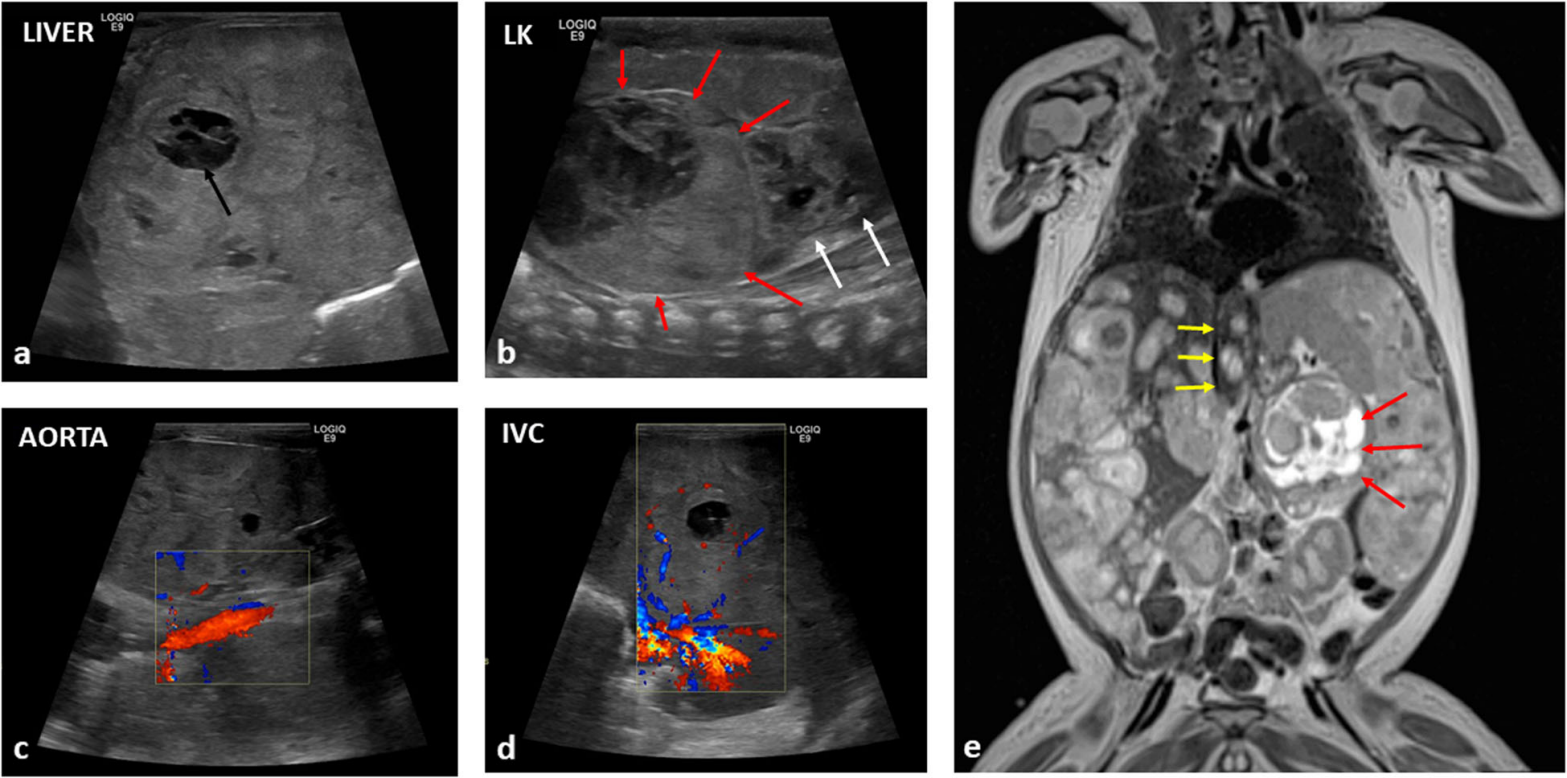
A newborn boy diagnosed with high risk, neuroblastoma at birth, presenting with abdominal distension, vomiting and discomfort. **a** Longitudinal ultrasound image of the right lobe of the liver demonstrates heterogenous internal echotexture with the impression of numerous mass-like lesions, some appearing necrotic (black arrow). **b** A longitudinal view of the left flank reveals a large suprarenal mass (red arrows), causing inferior displacement of the left kidney (white arrows). **c** Longitudinal views of the aorta demonstrates patency, although those of the (**d**) inferior vena cava only reveal scant flow within the liver. **e** Coronal T2-weighted MR imaging subsequently confirms the findings, demonstrating the left suprarenal mass (red arrows) with multiple diffuse metastases within the liver. The upper abdominal inferior vena cava is compressed (yellow arrows) putting the patient at risk of abdominal compartment syndrome. The patient also suffered from respiratory compromise from the enlarged liver hindering diaphragmatic movement, hepatic failure, obstructive jaundice and coagulopathy. As a secondary consequence of the large tumour bulk at diagnosis, the patient was also treated for tumour lysis syndrome after initiation of chemotherapy

Mass effect on the diaphragm from an intra-abdominal mass may cause splinting of the diaphragms, restricted self-ventilation and poor gaseous exchange. Causes can include gross hepatomegaly from primary hepatoblastoma or metastases in stage MS neuroblastomas which may require urgent intubation and ventilatory support (Fig. 11). Ascites associated with primary, typically non-capsulated masses is often a contributing factor, with some relief provided by immediate percutaneous drainage.

## Abdominal emergencies

### Abdominal compartment syndrome

Abdominal compartment syndrome is a potentially life-threatening complication carrying a mortality rate of up to 60% [52, 53]. The underlying mechanism is one where an increase in abdominal compartment volume exceeds the relative expansion capacity of the abdominal wall. This leads to inadequate end-organ perfusion, organ dysfunction and in extremis, organ failure. Splinting of the diaphragm and pulmonary atelectasis are often concomitant findings.

**Fig. 12:**
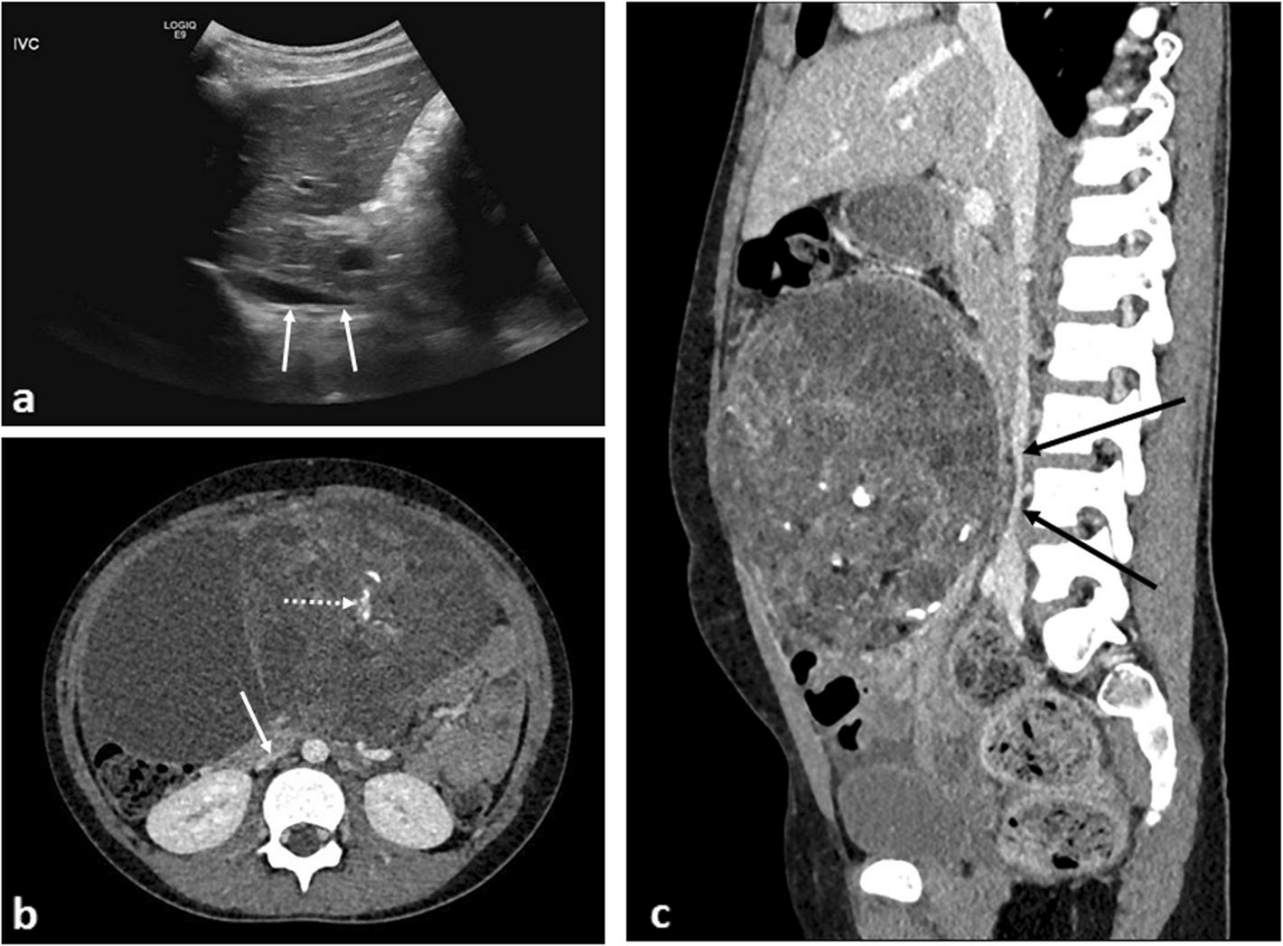
An 8-year-old girl with increasing abdominal distension secondary to a malignant germ cell tumour (MGCT). **a** A sagittal abdominal ultrasound image demonstrates tapering of the upper abdominal inferior vena cava (IVC) (white arrows). **b** Axial contrast-enhanced CT image at the level of the kidneys demonstrates flattening of the IVC (arrow). There is also a large heterogenous mass in the anterior abdomen containing internal foci of calcification (dashed arrow). **c** Sagittal CT image of the IVC reveals demonstrates compression of the vessel (black arrows), again placing the patient at risk of poor central venous return

The diagnosis is clinical; however, by identifying venous compression, particularly IVC obstruction on either an abdominal ultrasound or portal venous phase, post-contrast CT study, the radiologist can help raise awareness [54]. Surgical referral is essential and emergency decompressive laparotomy may be required. Possible aetiologies include any cause of a rapid increase in abdominal contents such as large (bilateral) Wilms tumours [55], rapidly proliferating acute lymphoblastic leukaemic infiltrates [56], giant ovarian masses [57] (Fig. 12) and ascites [58].

### Bowel obstruction

**Fig. 13:**
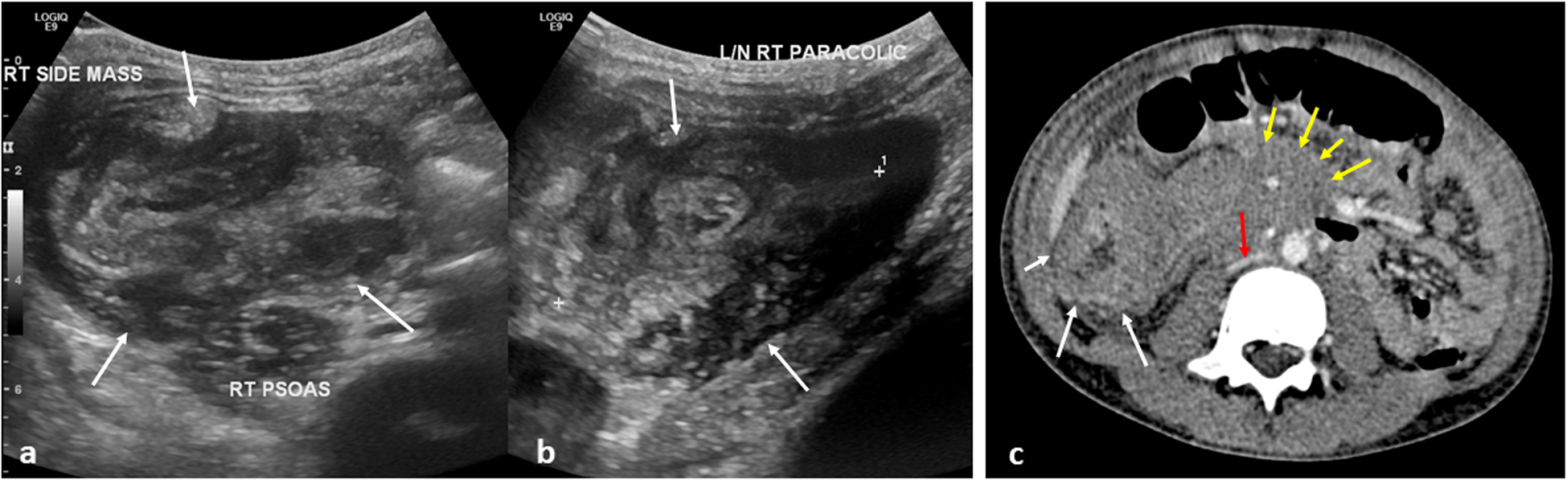
A 10-year-old boy with B-cell non-Hodgkin’s lymphoma, presenting with abdominal pain. **a**, **b** Initial transverse and longitudinal ultrasound views of the right paracolic gutter demonstrated a large complex mass (white arrows), suspected to represent an intussusception. **c** Axial contrast-enhanced CT of the abdomen, confirmed the finding of the intussusception (white arrow) with adjacent areas of inflamed and matted bowel loops, likely to represent an infiltrative process such as lymphoma. Further areas of extensive lymphadenopathy, predominantly in the pelvis and around the mesenteric vessels (yellow arrows) with compression of the IVC (red arrow), were also noted as ileo-colic intussusception. Given the age, size of the patient and subacute clinical history, air-reduction enema was deemed inappropriate and the patient underwent a surgical reduction of the intussusception

Bowel obstruction is frequently caused by intussusception. Whilst relatively common in healthy children with median age at presentation of 8 months [59], occurrence in children over 2 years of age should raise concerns for an underlying neoplastic lead point such as Burkitt’s lymphoma (where intussusception is the primary presentation in 18% of cases) [60] (Fig. 13). Ultrasound is the primary modality for identifying an intussusception and it may also demonstrate the lead point such as diffuse asymmetric bowel wall thickening, a tumour or lymphadenopathy. In such cases, hydrostatic or air enema reduction should be avoided as this is frequently unsuccessful and may have a higher likelihood of complication, such as bowel perforation.

### Bowel perforation

**Fig. 14:**
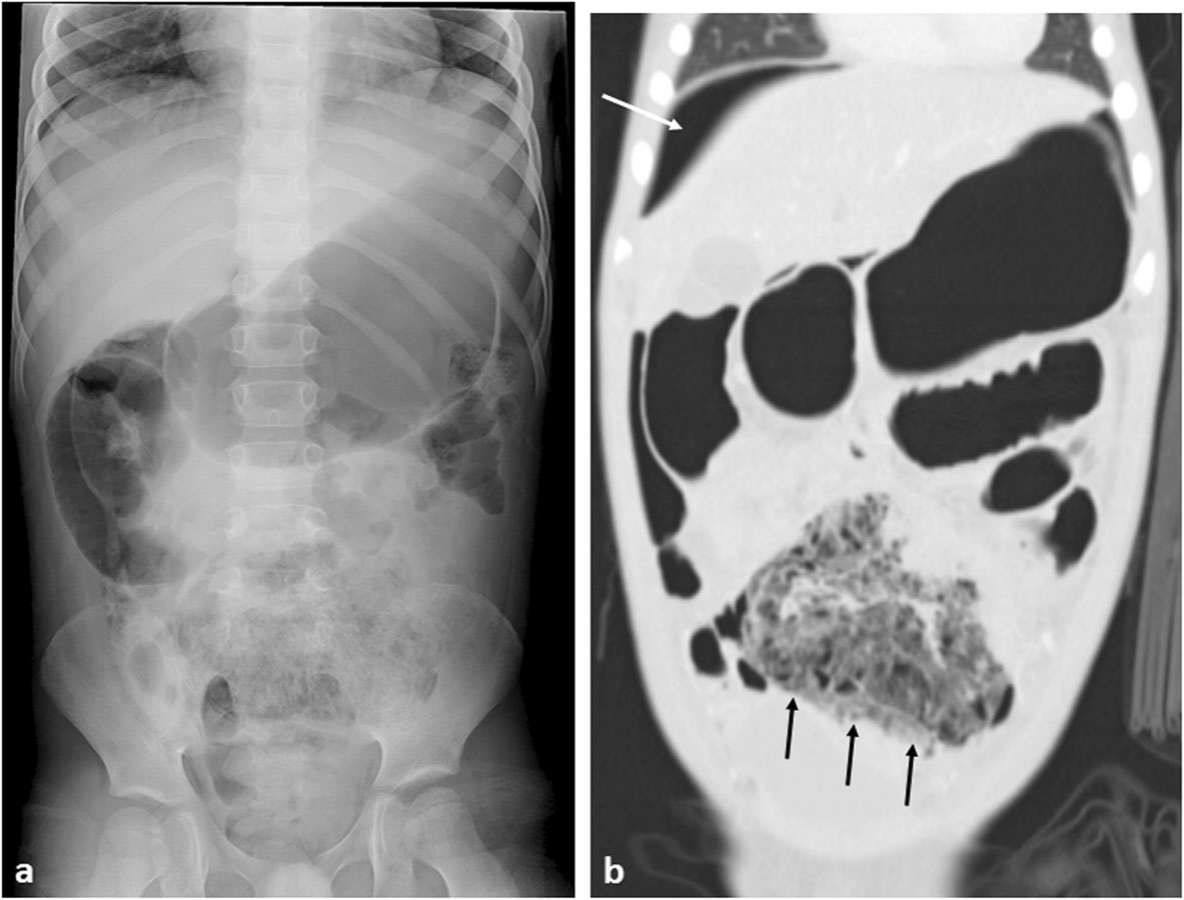
A 6-year-old boy with small bowel Burkitt’s lymphoma. **a** Abdominal plain radiograph demonstrates gastric and small bowel dilatation with a mottled gas appearance within the pelvis. **b** Coronal CT imaging with lung windows, demonstrates free intra-abdominal gas, best seen superior to the liver (white arrow), with proximal bowel obstruction and perforation of a thick-walled ileal mass (black arrow)

Bowel perforation may occur secondary to bowel obstruction, intestinal tumour infiltration (Burkitt’s lymphoma commonly (Fig. 14) [61]) or after intensive radiation and chemotherapy. Imaging with an erect or cross table lateral chest or abdominal radiograph or abdominal ultrasound may reveal pneumoperitoneum.

### Obstructive jaundice

**Fig. 15:**
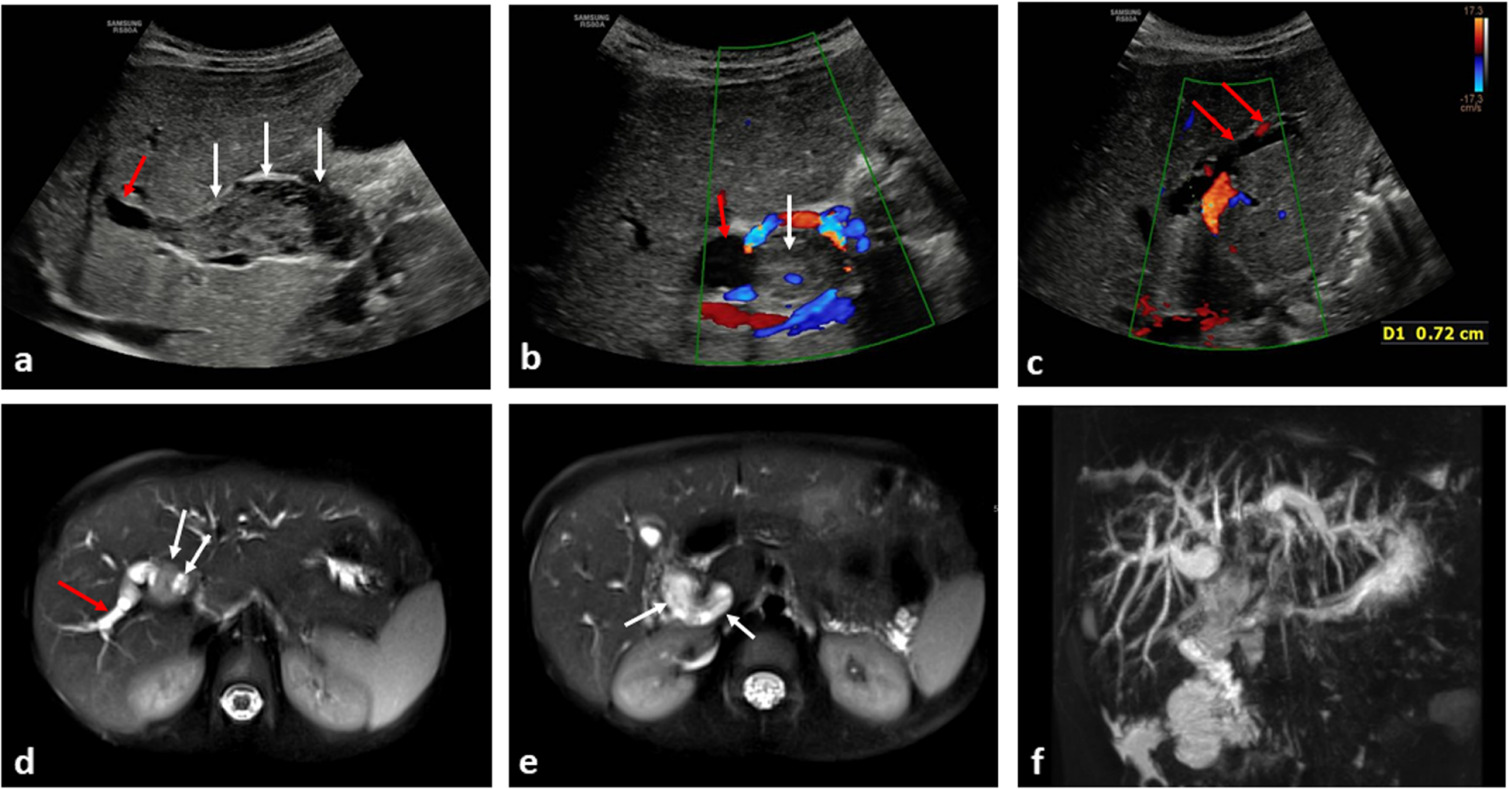
A 5-year-old boy presenting with jaundice from biliary rhabdomyosarcoma. **a** Longitudinal and (**b**, **c**) transverse ultrasound images of the liver at diagnosis, demonstrates a heterogenous mass (white arrows) occupying and distending the common bile duct with downstream intrahepatic biliary dilatation (red arrow). **d**, **e** Axial T2-weighted MRI images through the liver confirm the intra-biliary mass (white arrows), with dilatation of right-sided intrahepatic bile ducts (red arrow). **f** The intrahepatic biliary dilatation is best demonstrated on the 3D-maximum intensity projection (MIP) image of the MRCP (MR cholangiopancreatography)

**Fig. 16:**
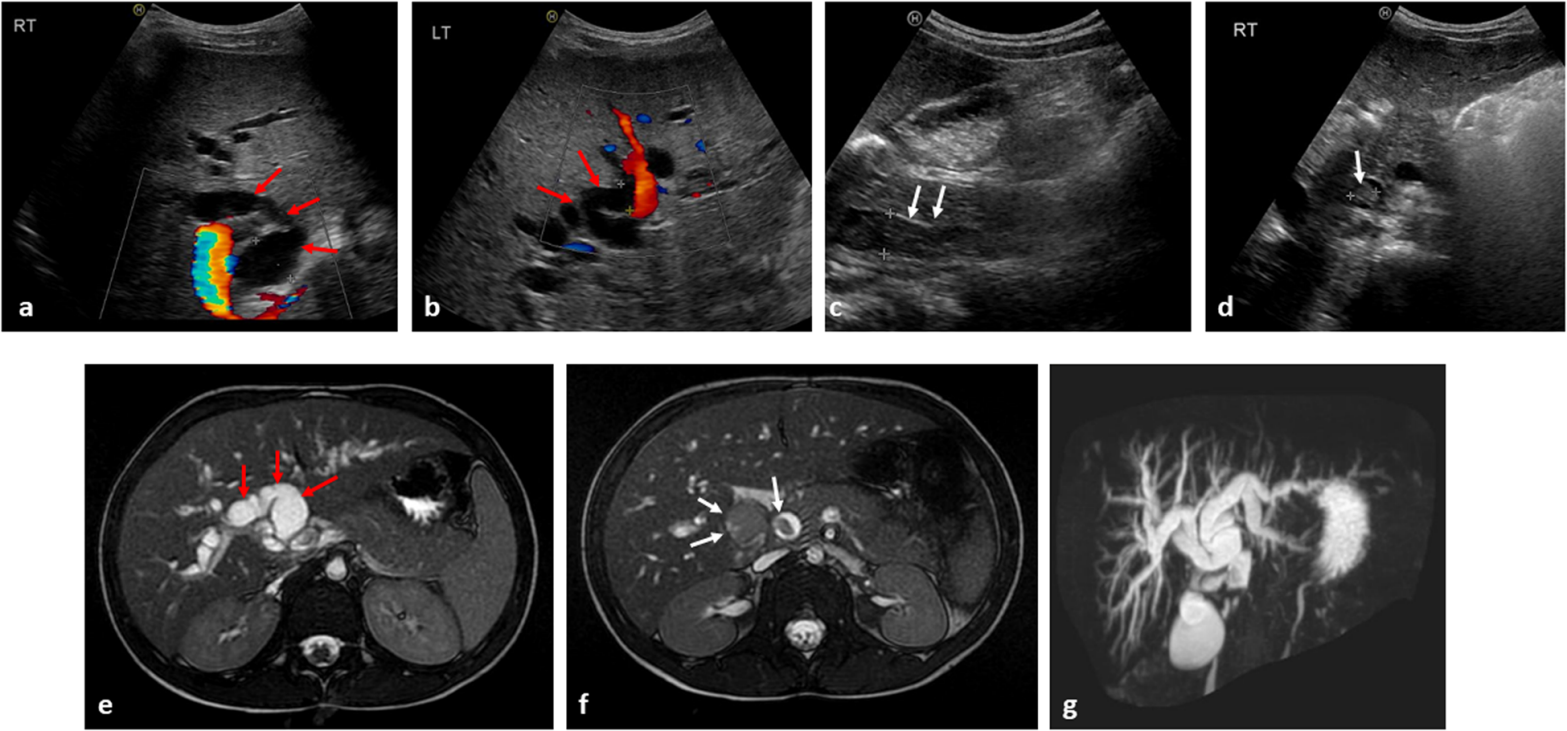
A 6-year-old girl, also presenting with jaundice from a biliary rhabdomyosarcoma. **a**, **b** Transverse ultrasound images at diagnosis reveal marked intrahepatic biliary ductal dilatation (red arrows) measuring > 1 cm in diameter within both the right and left hepatic lobes. **c**, **d** Upon careful assessment of the common bile duct to its origin, a homogenous intra-ductal mass is identified (white arrows). **e**, **f** Axial T2-weighted MRI images through the liver, subsequently confirm the intrahepatic ductal dilatation (red arrows), with intra-ductal mass (white arrows) in keeping with the biliary rhabdomyosarcoma**. g** The 3D-MIP from the MRCP again helps to demonstrate the extent of the intrahepatic biliary ductal dilatation. In this clinical scenario, compared to the images of Fig. 15, the intra-ductal mass is harder to visualise on ultrasound without careful assessment of the common bile duct to its origin, highlighting the importance of careful assessment and patience to ensure all pertinent findings are identified

Biliary rhabdomyosarcoma (RMS) is a rare primary malignancy, but the most likely primary tumour to cause biliary tract obstruction, and can be accompanied by abdominal distension and hepatomegaly [62, 63]. Occasionally, it may be misinterpreted as a hepatic mass such as a hepatoblastoma, which can also present with jaundice [64] or as an undifferentiated embryonal sarcoma due to the shared histopathological similarities (although embryonal sarcomas rarely cause biliary obstruction [65]). Lymphadenopathy at the porta hepatis or a pancreatic head mass due to pancreatoblastoma may be large but, again, seldom cause biliary dilatation (Figs. 15 and 16).

The diagnosis of biliary RMS remains challenging and first-line imaging is primarily focussed on ultrasound identification of biliary dilatation, with location of a soft tissue mass, frequently within the common bile duct [66]. Further imaging with MRI and MRCP sequences help to identify the primary mass and potential hepatic metastases [67]. The majority of such lesions are initially treated with chemotherapy (given their chemosensitivity), surgical interventions may be required in cases of relapse or where hepatic transplantation is considered [68, 69].

### Urinary tract obstruction

**Fig. 17:**
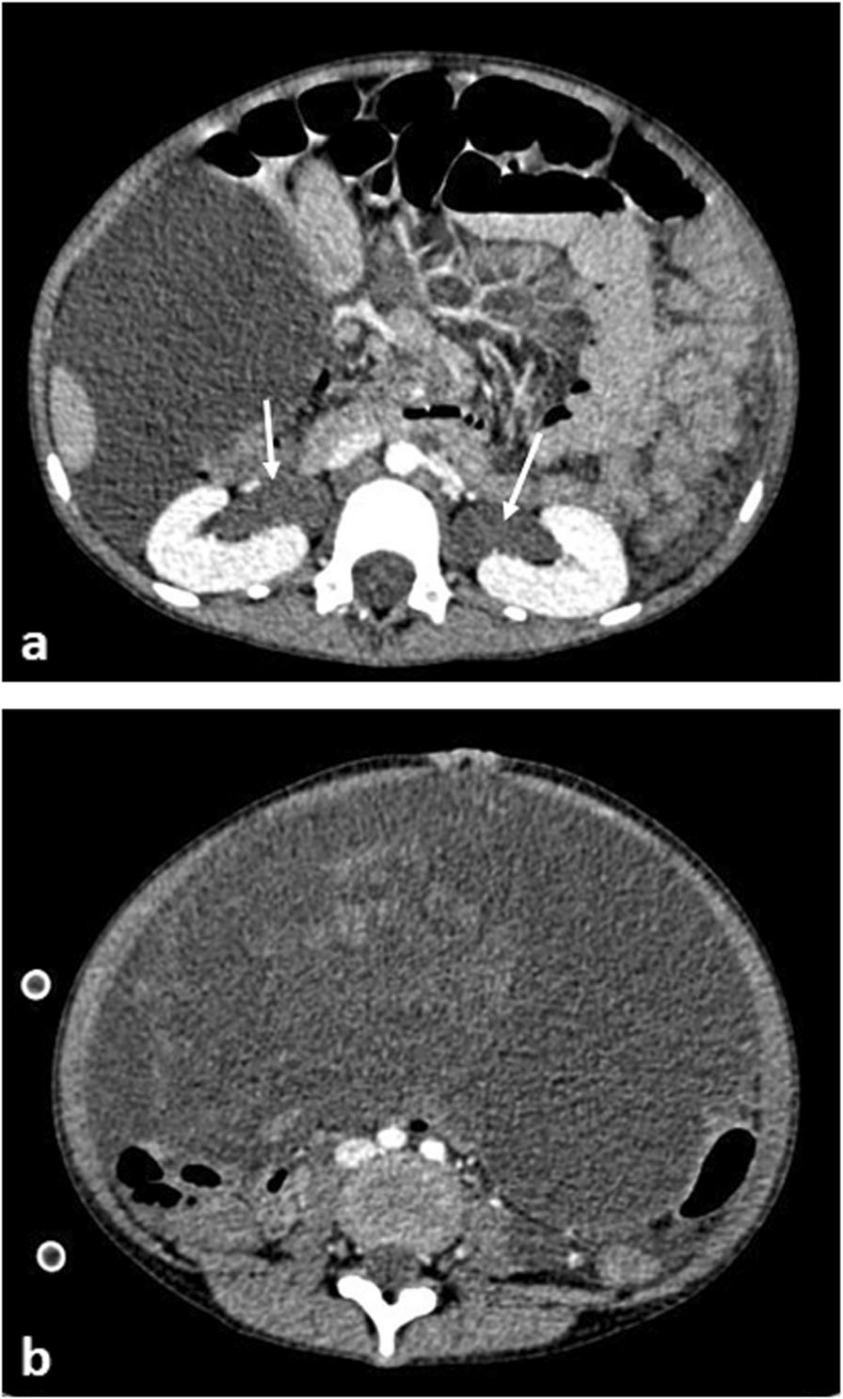
An 8-year-old girl with large Sertoli Leydig cell ovarian tumour arising from the right ovary. **a** Axial contrast-enhanced CT of the upper abdomen demonstrates bilateral hydronephrosis (white arrows) secondary to (**b**) the large intra-abdominal mass, which was compressing the distal ureters (not visible in image)

Pelvic masses such as rhabdomyosarcomas, neuroblastomas, sacrococcygeal teratomas and germ cell ovarian tumours in girls may present with distal ureteric obstruction and subsequent upper urinary tract dilatation (Fig. 17). Mass affect from enlarged lymph nodes in lymphoma may be seen in up to in 20% of cases. Finally, any large intra-renal mass may obstruct the urinary collecting system via invasion or mass effect. In the majority of cases, urinary tract obstruction resolves after treatment, with only a minority (13% [54]) requiring intervention. Ureteric stenting is preferred over percutaneous nephrostomy, due to the inherent lower risk of infection. Ultrasound is a reliable first-line modality for assessment of the urinary tract and pelvic masses, which can ultimately be further characterised with MRI.

## Haematological and vascular emergencies

### Venothromboembolism

**Fig. 18:**
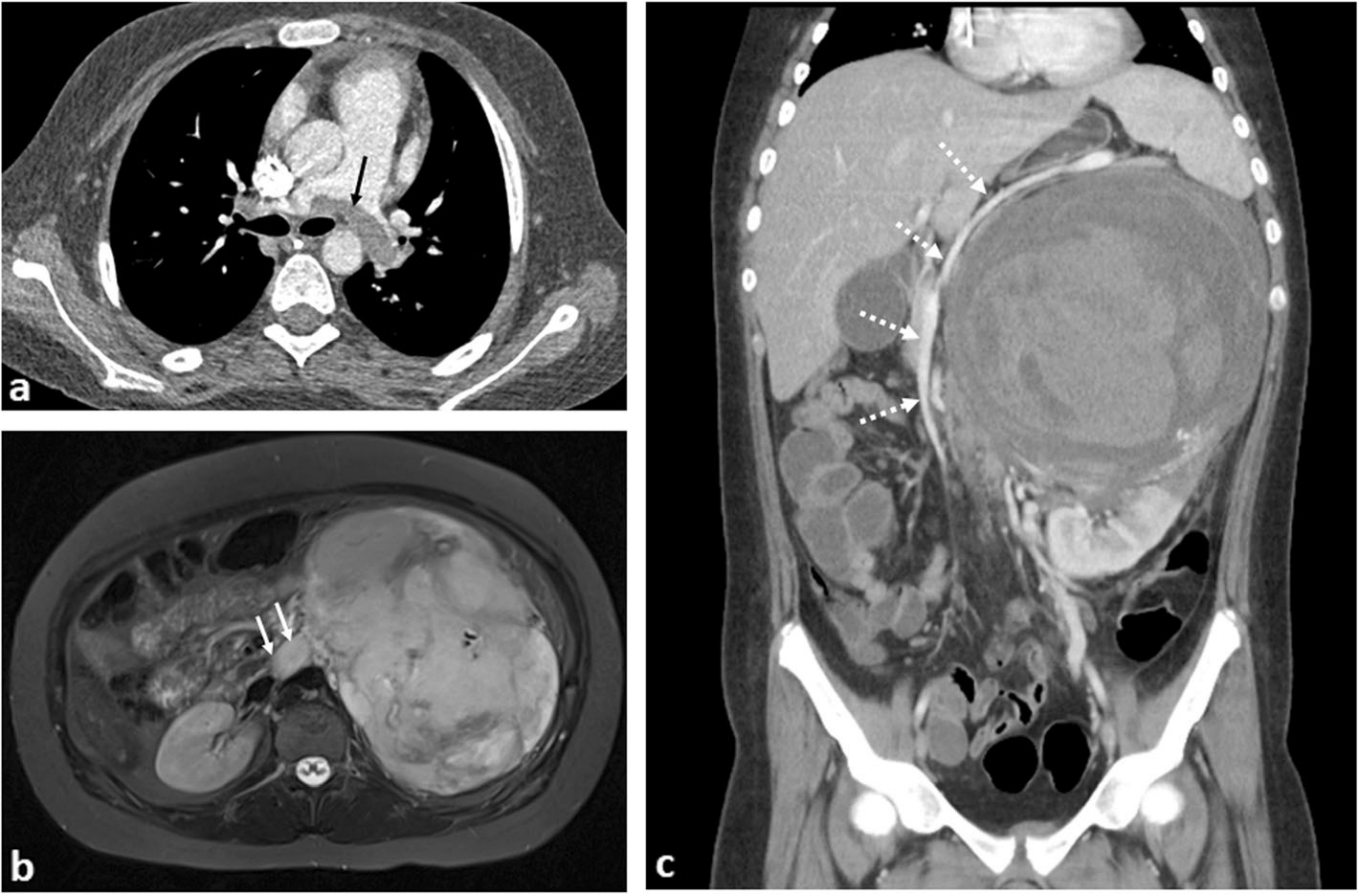
A 13-year-old girl with left-sided Wilms’ tumour and saddle pulmonary embolus. **a** Axial computed tomographic pulmonary angiogram (CTPA) demonstrates a saddle pulmonary embolus occupying the right and left pulmonary arteries (black arrow). **b** Axial T2-weighted fat-saturated MR image reveals a left renal mass with tumour thrombus occupying the entire left renal vein (white arrow). **c** Coronal contrast-enhanced CT image of the abdomen shows the heterogeneously enhancing left renal mass, with internal haemorrhage. There is right-sided displacement of the IVC with the splenic vein (dashed arrows) stretched and draped over the mass

Whilst a rare complication (with respect to adults), the incidence of thromboembolism (both deep venous thromboembolism and pulmonary emboli) in children with cancer is still higher than that seen within their healthy counterparts. One British study estimated the thrombosis risk at 1.52 per 1000 person-years for children with cancer (95% CI = 0.57–4.06) versus 0.06 per 1000 person-years (95% CI = 0.02–0.15) in children without cancer [hazard ratio of 28.3 (95% CI = 7.0–114.5)] [70]. Overall, it is more commonly associated with complications from therapy (i.e., indwelling central venous line, steroid or L-asparaginase treatment [71]). Primary malignancies causing increased hyperleucocytosis (e.g. in ALL), reduced mobility (e.g. soft tissue and bone sarcomas [72]) or associated with tumour thrombosis (4–8% of patients with Wilms tumours [34] (Fig. 18)) account for the remainder of cases.

Doppler ultrasound imaging of the extremities, including venous compression techniques should be adopted to identify suspected limb thromboembolism, whilst CT pulmonary angiography (CTPA) is suitable for suspected pulmonary emboli. Contrast-enhanced CT head for venous sinus thromboses have been discussed earlier in this article. At present, there are no specific guidelines for thromboprophylaxis treatment in children with cancer. It is therefore even more important for radiologists to be astute to the presence of potential thromboembolism in children.

### Haemorrhage and disseminated intravascular coagulopathy

**Fig. 19:**
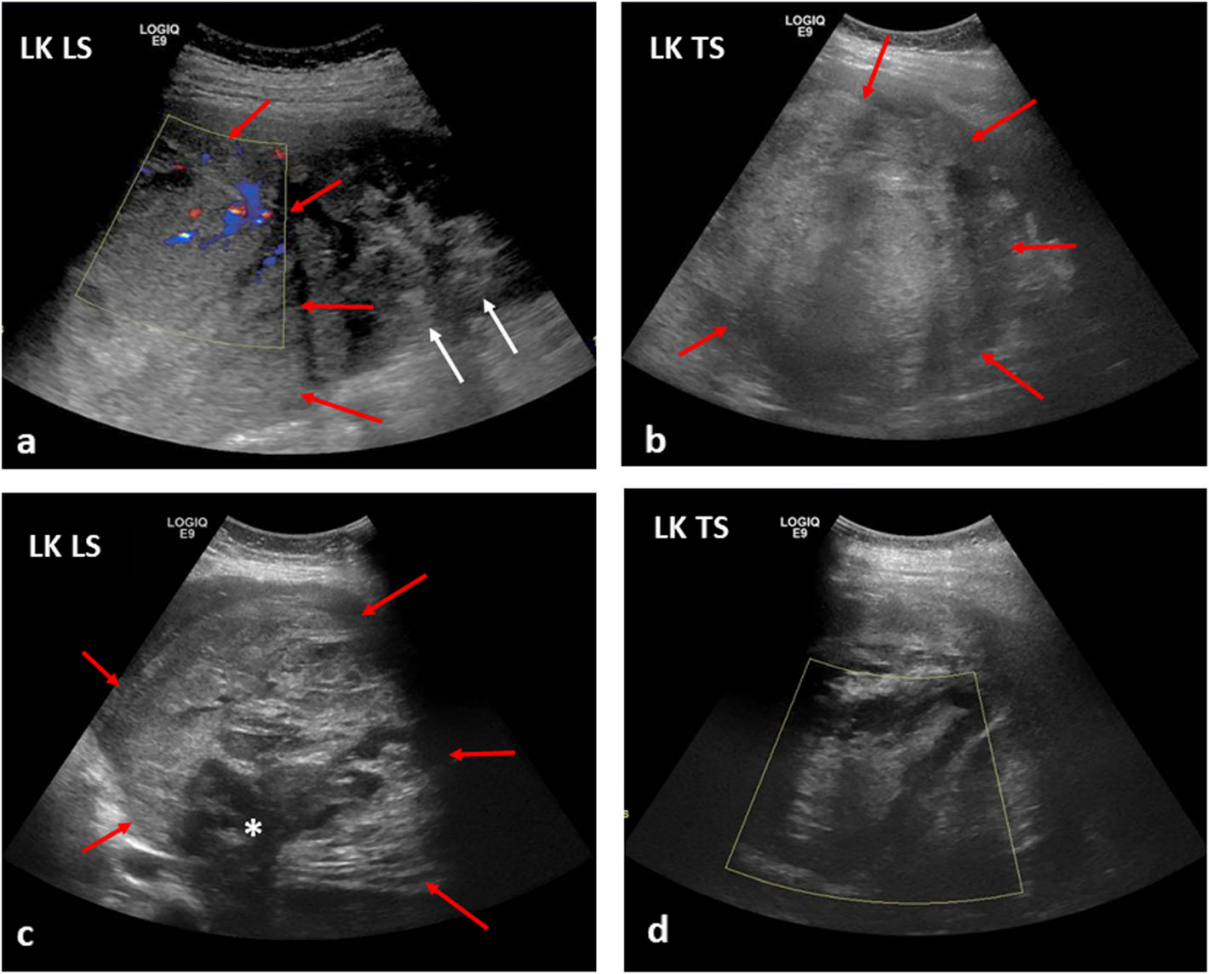
The same 13-year-old girl as in Fig. 18, with subsequent internal haemorrhoage within a left-sided Wilms’ tumour. **a**, **b** At diagnosis, the initial longitudinal (LS) and transverse (TS) ultrasound images of the left kidney (LK) reveal a large echogenic mass (red arrows) in the upper pole of the kidney, with some preserved renal tissue in the lower pole (white arrows). Some scant internal colour Doppler flow is noted within the mass. **c**, **d** A month later, after a sudden drop in the haemoglobin levels accompanied with intense abdominal pain, the ultrasound images of the left kidney reveal a larger, more heterogenous mass (red arrows) with some internal necrosis (white asterisk) and no internal colour Doppler flow in keeping with a large haematoma. It was not possible to clearly delineate the left renal vein at either the initial or subsequent ultrasound studies

**Fig. 20:**
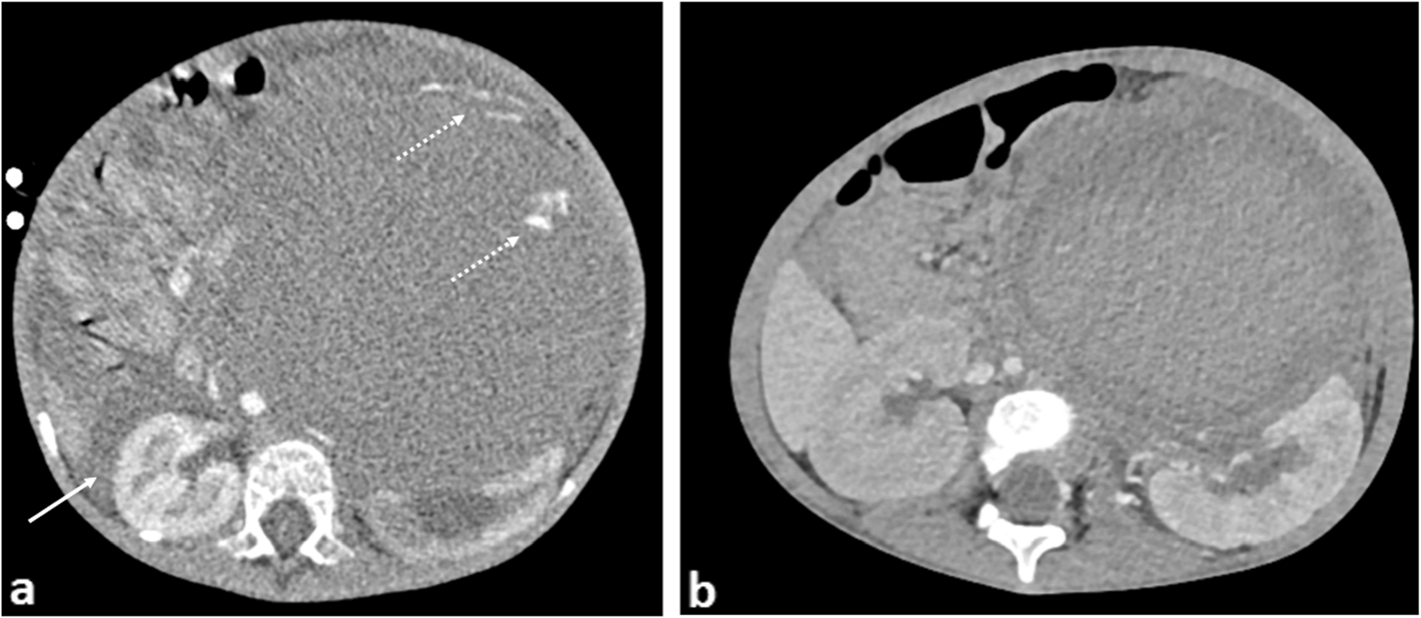
A 3-year-old girl with stage 4 neuroblastoma and acute intra-tumoural haemorrhage. **a** Axial CT imaging obtained during an episode of acute abdominal pain and drop in haemoglobin shows a large left suprarenal mass with right paracolic free fluid (white arrow) and a blush of contrast within the peripheral left aspect of the mass (dashed arrow) in keeping with active contrast extravasation. **b** A repeat CT image obtained after vascular embolisation 1 month later reveals reduction in the size of the mass with organisation of the haematoma. The degree of left renal hydronephrosis also appears reduced

Disseminated intravascular coagulopathy (DIC) is characterised by excessive activation of blood coagulation and consumption of clotting factors. It is found in children with disseminated metastases or secondary to AML [73], where it can cause early death [74]. DIC may also result from a consumptive coagulopathy (Kasabach-Merritt syndrome [75]) associated with vascular tumours such as Kaposiform haemangioendothelioma. Multiphase, intravenous contrast-enhanced CT imaging in the acute scenario can help to confirm the location of the acute haemorrhage for embolization, although treatment is largely supportive (Figs. 19 and 20).

Whilst a pre-contrast CT of the affected body part may be routinely acquired in adult radiology, this is generally avoided in paediatric imaging with only the arterial and portal venous phase imaging being sufficient [76].

### Hypertension

**Fig. 21:**
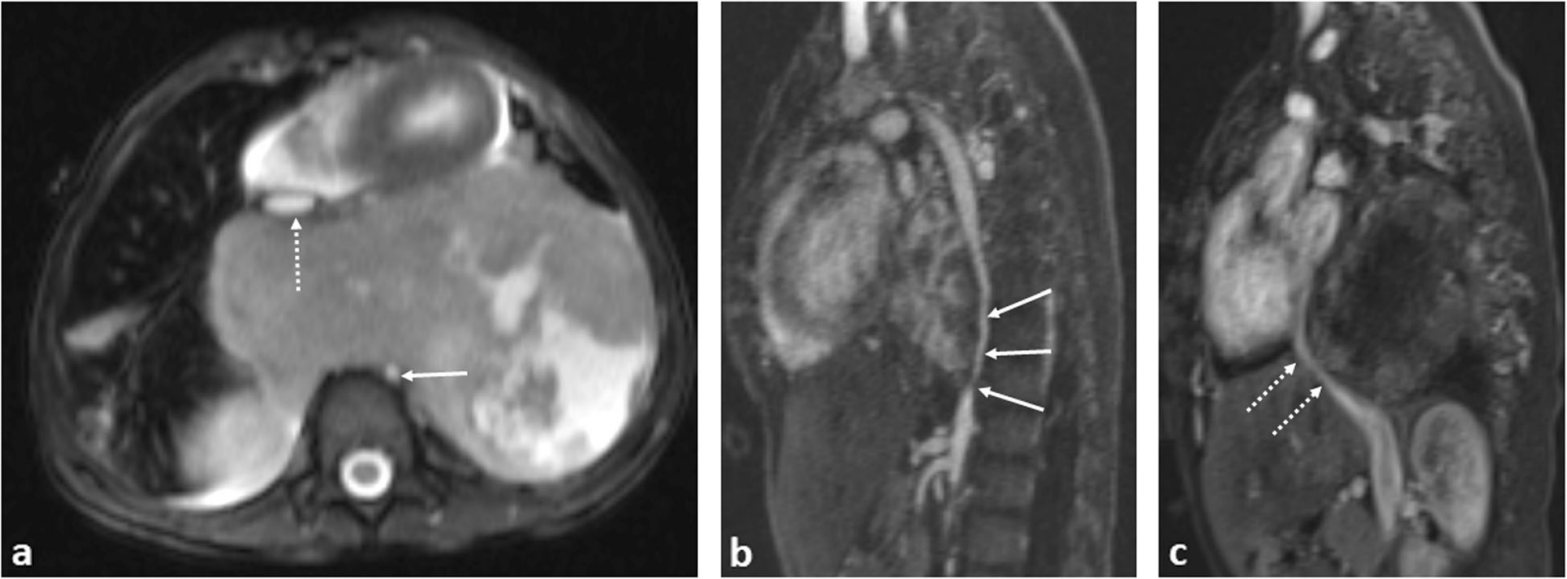
Three-year-old boy with a large posterior mediastinal malignant nerve sheath tumour causing marked hypertension due to descending thoracic aortic compression. **a** Axial T2-weighted MRI image showing severe narrowing of the descending thoracic aorta (solid arrow) and anterior displacement of the IVC (dashed arrow). Sagittal post-contrast T1 images highlight the descending thoracic aortic (**b**) and upper abdominal IVC (**c**) compression, respectively. Due to the extensive nature of the mass, surgical resection was not through to be appropriate. The patient has subsequently undergone several cycles of chemotherapy and balloon dilatation of the thoracic aorta accompanied by a slow, but gradual improvement in symptoms and size of tumour

End-organ damage or haemorrhagic stroke secondary to refractory hypertension may be the presenting feature of some paediatric malignancies. These can be biochemical in nature (such as from excessive catecholamine production in neuroblastomas or phaeochromocytomas) or from mass effect on the aorta or renal arteries (Fig. 21).

Whilst vascular compression from a mass may be investigated with ultrasound examination and spectral Doppler analysis, definitive characterisation with cross-sectional imaging and angiography should be performed for surgical planning and/or endovascular intervention.

## Conclusions

Familiarity with life-threatening oncological emergencies is essential in contributing to the continued low rates of mortality and morbidity in children. Given that most children suffering from an acute complication may not initially present to a specialist centre, it is the responsibility for all general radiologists to have a basic awareness of such cases. In this article, we have provided an overview of the various ways in which oncological complications manifest and the main imaging considerations. Ultimately a multidisciplinary approach with good team communication and prompt action are all required to ensure the best imaging and treatment strategies going forward.

## Data Availability

Data sharing is not applicable to this article as no datasets were generated or analysed during the current study.

## Abbreviations

3D: three dimensional
ALL: Acute lymphoid leukaemia
AML: Acute myeloid leukaemia
CSF: Cerebrospinal fluid
CT: Computed tomography
CVST: Cerebral venous sinus thrombosis
DIC: Disseminated intravascular coagulopathy
ETMR: Embryonal tumours with multi-layered rosettes
ICP: Intracranial pressure
MIP: Maximum intensity projection
MPNST: Malignant peripheral nerve sheath tumour
MRCP: Magnetic resonance cholangiopancreatography
MRI: Magnetic resonance imaging
MRT: Malignant rhabdoid tumour
SVC: Superior vena cava
